# Marginal Discrepancy and Internal Fit of 3D-Printed Versus Milled Laminate Veneers: An In Vitro Study

**DOI:** 10.3390/jfb15110338

**Published:** 2024-11-11

**Authors:** Arwa Daghrery, Honey Lunkad, Khalid Mobarki, Majed Alhazmi, Hussain Khubrani, Thilla Sekar Vinothkumar, Eman Jabarti

**Affiliations:** 1 Department of Restorative Dental Sciences, Division of Operative Dentistry, College of Dentistry, Jazan University, Jazan 45142, Saudi Arabia or vinothkumar_ts@yahoo.com (T.S.V.); 2 Department of Prosthetic Dental Sciences, College of Dentistry, Jazan University, Jazan 45142, Saudi Arabia or drhoney_82@yahoo.co.in (H.L.); 3 College of Dentistry, Jazan University, Jazan 45142, Saudi Arabia; 201805592@stu.jazanu.edu.sa (K.M.); 201805599@st.jazanu.edu.sa (M.A.); 201800023@st.jazanu.edu.sa (H.K.); 4 Dental Department, Dr. Sulaiman AlHabib Medical Group, Riyadh 2512, Saudi Arabia; eman.jabarti@drsulaimanalhabib.com

**Keywords:** dental veneers, cone-beam computed tomography, 3D printing, CAD-CAM, digital technology

## Abstract

The clinical success of laminate veneers depends upon their marginal discrepancy (MD) and internal gap (IG). This study aims to compare and evaluate the MD, overall discrepancy (OD), absolute marginal discrepancy (AMD), and IG of 3D-printed (Varseosmile TrinQ and Varseosmile Crown Plus) and computer-aided design and manufacturing (CAD/CAM)-milled (Brilliant Crios) laminate veneers using cone-beam computed tomography (CBCT). Thirty maxillary central incisors were prepared and divided randomly into three groups (*n* = 10). All teeth were scanned for veneer fabrication: Group PVT teeth with 3D-printed Varseosmile TrinQ, Group PVC teeth with 3D-printed Varseosmile Crown Plus, and Group BCM teeth with Brilliant Crios milled veneers. The specimens with respective veneers were scanned using CBCT, and the sectional images were measured for IG, MD, OD, and AMD. The mean values for MD recorded were 0.27, 0.31, and 0.40 for PVT, PVC, and BCM respectively). The mean values for IG recorded were as follows: PVT group—0.24, PVC group—0.28, and BCM group—0.39, and those for OD were as follows: PVT—0.22, PVC—0.32, and BCM—0.41. Intragroup significance was observed for IG and OD (*p* = 0.001). Findings revealed that milled veneers have a higher IG and MD than 3D-printed veneers, making them less clinically acceptable.

## 1. Introduction

Laminate veneers are commonly used in cosmetic dentistry to enhance esthetics and correct dental defects [1,2]. They are among the most used treatment options in esthetic dentistry owing to their biocompatibility, strength, and esthetic values [3]. The most common and reliable technique for fabricating laminate veneers is subtractive milling through computer-aided design and computer-aided manufacturing (CAD/CAM). However, this technique has various drawbacks, such as its high cost, amount of wastage, complex geometries, and wear on milling burs [2,4]. To overcome these drawbacks, additive manufacturing was introduced, enabling the faster fabrication of thin restorative materials and complex structures with high accuracy [5,6,7]. Previous studies have reported that laminate veneers fabricated using additive and subtractive techniques exhibit comparable biocompatibility and mechanical properties, with no significant differences [8,9,10].

The long-term clinical performance of laminate veneers depends on factors such as marginal and internal adaptation [10]. The marginal discrepancy or gap is categorized into vertical, horizontal, and absolute marginal discrepancy. A marginal discrepancy is defined as a vertical distance between the finish line of the preparation and the cervical margin of restoration [11,12]. This could be measured under high magnification with a stereomicroscope [1]. A marginal gap of 50 to 120 µm is considered as clinically acceptable for veneer restoration [13]. Insufficient sealing between restorations and preparations may lead to the leakage of oral fluids along the interfaces between veneers and teeth and can result in cement dissolution, postoperative sensitivity, marginal discoloration, and recurrent caries [8]. Hence, measuring marginal discrepancy and internal adaptation is important to record the long-term success of restorations.

There are various veneer materials available; however, in the current study, the authors utilized three: VarseoSmile TriniQ, VarseoSmile Crown Plus, and Brilliant Crios. Both VarseoSmile TriniQ and VarseoSmile Crown Plus are ceramic-filled, acrylate-based hybrid materials suitable for temporary and permanent restorations [14]. Brilliant Crios, on the other hand, is a reinforced composite bloc for permanent, indirect restorations using a CAD/CAM grinding process [9]. VarseoSmile TriniQ and VarseoSmile Crown Plus are light-curing, free-flowing materials primarily used for 3D-printed crowns, inlays, onlays, and veneers [14]. VarseoSmile TriniQ is ideal for temporary or less-load-bearing restorations that require flexibility and ease of printability, while VarseoSmile Crown Plus is better suited for permanent restorations due to its durability and long-term success [14]. Brilliant Crios is excellent for CAD/CAM, offering high-quality esthetics and resilience, with a ceramic-resin hybrid suitable for long-term wear [9].

Various techniques have been utilized to measure the marginal discrepancy and internal gap of restorations, such as the direct technique, sectioning method, profilometry, cone-beam computed tomography (CBCT), micro-CT technique, and silicone replica technique [15,16,17,18]. Each method has advantages and disadvantages. The literature suggests that 3D imaging techniques accurately measure the internal gap and marginal discrepancy of restorations [19]. Specifically, 3D images can be reconstructed to measure marginal discrepancy and the internal gap in various directions and sections, making it possible to assess multiple reference points simultaneously, which can eventually help evaluate the distance between restoration and prepared teeth. Studies have suggested that CBCT imaging provides high-quality images for measuring and evaluating marginal discrepancy and internal gaps. This imaging technique is non-destructive but requires special care to avoid errors [20].

Limited evidence in the literature is available on the marginal and internal gaps of 3D-printed laminate veneers. A study published comparing the mechanical properties of veneers prepared by 3D printers and CAD/CAM measured no statistical significance [21]. Another study reported the high biocompatibility of 3D-printed crowns [6]. A study on 3D-printed zirconia veneers reported the clinically acceptable internal adaptation of 3D-printed crowns compared to the conventional technique [22]. Reports in the literature have shown controversial findings on the marginal discrepancy and internal gap of laminate veneers prepared by 3D printing compared to conventional and CAD/CAM technologies. Moreover, there are limited studies evaluating MD and IG with CBCT analysis. Therefore, this in vitro study evaluated and compared the marginal adaptation, marginal discrepancy, overall discrepancy, and internal gaps of two 3D-printed, and CAD/CAM-milled laminate veneers. The first null hypothesis of this study was that there is no significant difference in the marginal discrepancy, internal gap, or absolute marginal discrepancy between laminate veneers fabricated by additive and subtractive techniques. The second null hypothesis was that there is no significant difference in the marginal discrepancy, internal gap, or absolute marginal discrepancy between laminate veneers prepared with three different materials.

## 2. Materials and Methods

The internal review board of the College of Dentistry, Jazan University, approved the present in vitro study (Ref no. REC-45/05/896).

### 2.1. Study Design and Specimen Preparation

The sample size was calculated by utilizing the G*Power statistical power-analysis program for Windows (version 3.1.9.4). A total sample size of 30 sound human maxillary anterior teeth (*n* = 10 in each group) was sufficient to detect a large effect size (d) = 1.55, with an actual power (1-β error) of 0.8 (80%) and a significance level (α error) of 0.05 (5%). The inclusion criteria were sound human maxillary central incisors extracted for periodontal and orthodontic treatment. Deciduous, carious, and fractured teeth were excluded. The extracted teeth were collected and stored in distilled water for a week before the commencement of research. Each tooth was embedded in self-curing acrylic resin (1.6 cm in diameter and 2.0 cm in height), with the cementoenamel junction just exposed at the surface of the resin.

Standardized tooth preparation was carried out for all 30 samples by one experienced prosthodontist. An index was prepared using condensation silicone impression material (Silibest; BMS Dental, Capannoli, Italy) to control the depth of the preparation. A veneer preparation followed, beginning with the placement of deep orientation grooves using a three-wheel diamond depth cutter (LVS1 Veneer Depth Cutting Diamond Bur 834-021: Brasseler USA Dental, Savannah, GA, USA) which were then reduced using tapered round-ended diamond rotary instruments (FG and T&F hybrid points, Shofu Inc., Kyoto, Japan). The preparation design incorporated a butt joint incisal overlap with 1.5 mm of incisal reduction, excluding a palatal chamfer. Additionally, a 1 mm wide chamfer finishing line was extended interproximally and positioned equigingivally, along with a 1 mm labial reduction. The preparation was finalized using finishing stones (Shofu, Inc., Kyoto, Japan). These prepared teeth were then randomly divided into three groups of 10 each (Table 1): Group PVT—3D printed using Varseosmile TrinQ (Bego, Bremen, Germany), Group PVC—3D printed using Varseosmile Crown Plus (Bego), and Group BCM—milled with Brilliant Crios (Coltene Whaledent, Altstatten, Switzerland).

### 2.2. Digital Workflow

The prepared teeth were scanned using an Itero oral scanner (Itero element 2; Align Technology Inc., San Jose, CA, USA) to create a virtual die. The produced files were exported to a Standard Tessellation Language (STL) format, which was used to digitally design and fabricate the laminate veneers. Virtual images of the restorations were designed with a cement space of 40 µm.

The Digital Light Processing printer (Max UV, Asiga, Sydney, Australia) was used to fabricate 3D-printed laminate veneers (*n* = 10) at a wavelength of 405 nm, resolution of 50 µm, and printing speed of 0.25 mm/min at 23 °C per group. After the 3D printing procedure was completed, the laminate veneers were carefully removed from the machine with the help of a spatula. The veneers were cleaned in an ultrasonic bath (Foshan Adelson Medical Devices Co., Foshan, China) using 96% ethanol (Thermo-fisher Scientific, Riyadh, Saudi Arabia) for 480 s until all unpolymerized resin was removed and then gently air-dried.

For the CAD/CAM group (BCM), the STL files were transferred to the CAD nesting software (CEREC version 4.X; Dentsply Sirona, Bensheim, Germany) and wet-milled using a 5-axis milling machine (inLab MCX5; Dentsply Sirona) (*n* = 10). The milling settings were selected according to manufacturer instructions to produce Brilliant Crios (Coltene/Whaledent AG) milled veneers. All the prepared veneers were steam-jet cleaned and air-dried. Before further analysis, these specimens were evaluated for manufacturing defects. Any remnants detected were removed, and the surface was smoothed, except for incisal edges, to prevent errors during the seating procedure. All the veneers were carefully evaluated prior to CBCT scan for any visible defects (including porosities) but nothing was detected.

Subsequently, the external and internal surfaces of prepared restoration were post-cured under the Otoflash light-curing device (Bego). The prepared samples were subjected to 1500 flashes of ultraviolet lights at 10 Hz in an atmosphere containing nitrogen gas (1.0–1.2 bar).

All the laminate veneers were secured to the respective prepared tooth on cervical, distal, mesial, and palatal aspects with small pieces of adhesive tape prior to CBCT.

### 2.3. Marginal Discrepancy and Internal Gap Assessment by CBCT

The seated laminate veneers were scanned using CBCT (Kodak 9500 cone beam 3D; Carestream Health Inc., Rochester, NY, USA). Two parameters were considered to assess marginal discrepancy: marginal discrepancy (MD) and absolute marginal discrepancy (AMD). MD is the perpendicular distance from the internal surface to the preparation’s margin, measured from the cervical and incisal margins [1]. AMD is the distance from the internal coping margin to the prepared finish line, measured at the cervical and incisal margins [1]. Internal gap (IG) is the perpendicular distance from the internal surface of the coping margin to the axial wall of the tooth preparation [12].

**Figure 1:**
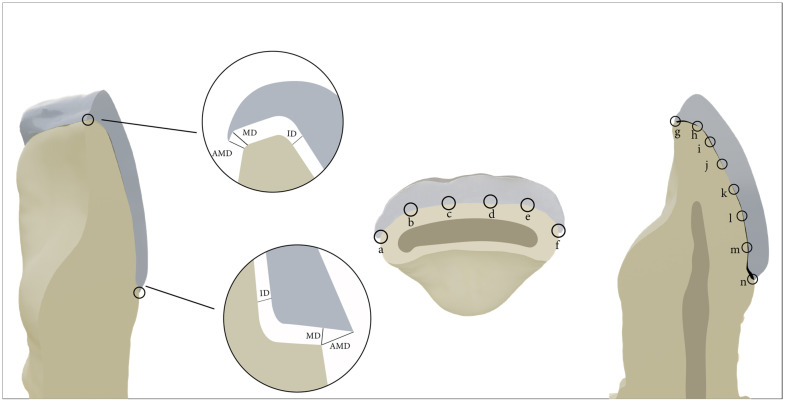
The points of marginal and internal discrepancy measurements in laminate veneers. AMD: the absolute marginal gap at the cervical margin; MD: marginal discrepancy; ID: internal discrepancy; points a, f, h, and m represent the marginal discrepancy and points b, c, d, e, i, j, k, and l represent the internal gap; point g represents the absolute marginal discrepancy at the incisal edge and point n represents the absolute marginal discrepancy at the cervical edge.

The prepared laminate veneers were organized in a circular pattern on the occlusal plate of the CBCT scanner. Axial and sagittal sections were used to measure the internal and marginal gaps of the prepared laminate veneer, as detailed in Figure 1.

The MD, IG, OD, and AMD at the cervical and incisal margins were measured using digital imaging processing software (3D module version 2.4 Kodak Dental Imaging software; Carestream Health Inc.). Each specimen was photographed using a USB digital microscope with a built-in camera (Scope Capture Digital Microscope, Guangdong, China) connected with an IBM compatible personal computer using a fixed magnification of 90× for the marginal gap and internal discrepancy.

### 2.4. Statistical Analysis

Statistical analysis was performed using SPSS software version 22 (IBM SPSS Statistics for Windows, version 22.0; IBM Corp, Chicago, IL, USA.). Numerical data were tested for normality and represented as the mean and standard deviation (SD). Given that the obtained data were normally distributed, a one-way analysis of variance (ANOVA) test was performed. A pairwise comparison was conducted using the post hoc Bonferroni test. To obtain the statistical significance among the variables, the *p*-value was set at <0.05.

## 3. Results

**Figure 2:**
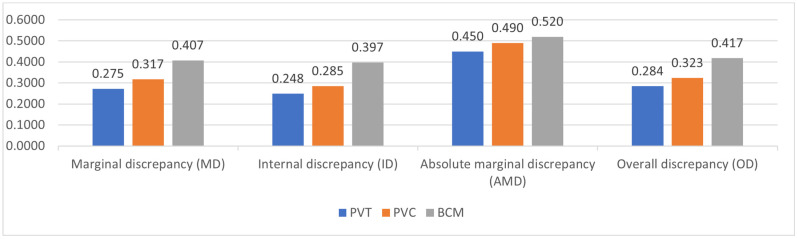
Comparison of marginal, internal, absolute, and overall discrepancy among groups.

**Figure 3:**
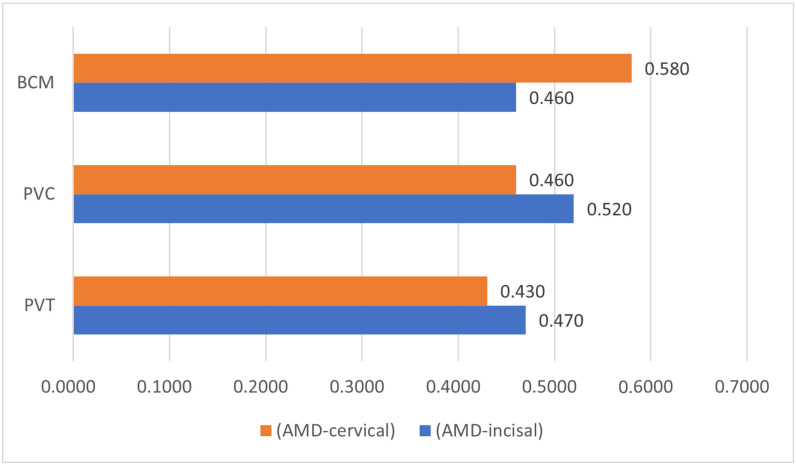
Bar chart representing absolute marginal discrepancy at cervical and incisal end among three groups.

The result of one-way ANOVA measured for comparison between the groups is represented in Table 2 and Figure 2. Statistical significance was reported among the two variables, the internal and overall discrepancies, within the three groups, with a *p*-value of 0.001. The mean values for internal discrepancy recorded in all three groups were as follows: PVT group—0.248 (SD 0.192), PVC group—0.285 (SD 0.22), and BCM group—0.397 (SD 0.050). The mean values recorded for overall discrepancy for the three groups were as follows: PVT—0.22 (SD 0.089), PVC—0.32 (SD 0.26), and BCM—0.41 (SD 0.034). However, there was no statistical significance between marginal and absolute marginal discrepancy among the three groups. Similarly, no statistical difference was reported among the groups in incisal and cervical absolute marginal discrepancies (Table 2 and Figure 3). Absolute marginal discrepancy was highest at the cervical edge of the veneer fabricated by the CAD/CAM procedure (0.50) and lowest for the PVT group (0.43). However, absolute marginal discrepancy at the incisal edge was highest for the veneers fabricated by 3D printing (Varseosmile Crown Plus), as compared to CAD/CAM. The lowest mean value was reported among the marginal discrepancy (0.22), internal discrepancies (0.24), and overall discrepancies (0.28) of veneers in the PVT group.

A post hoc Bonferroni test was performed for a pairwise comparison between the overall and internal discrepancies (Table 3). Group PVT and BCM (0.0001) and PVC and BCM (0.006) showed statistically significant internal discrepancies, with mean differences of −0.148 and −0.112, respectively. Statistical significance was also reported in the overall discrepancy between PVT and BCM (0.001), with a mean difference of −0.133. The Bonferroni test showed that the mean difference among the points was negative between the groups, indicating that the PVT group had the least internal discrepancy and overall discrepancy, followed by the PVC group and BCM group.

**Figure 4:**
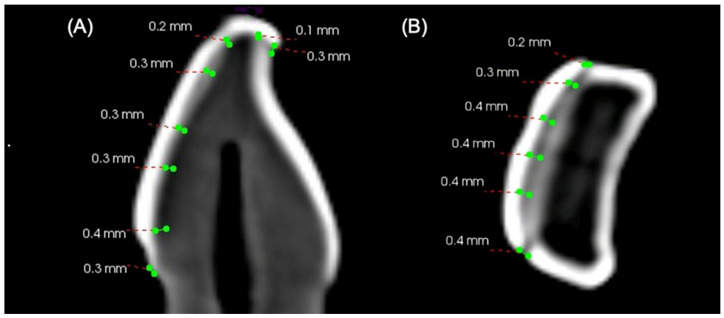
Representative CBCT sectional images representing measurements for internal gap and marginal discrepancy at incisal edge and cervical edge: (**A**) sagittal view and (**B**) axial view.

Figure 4 shows the CBCT analysis, representing the mean differences in marginal discrepancy and internal gap among the 14 points studied. A total of 60 images were taken, and the average internal gap and marginal discrepancy were compared and presented graphically (Supplementary Figure S1). The CBCT analysis reveals that laminate veneers prepared using 3D printers (*n* = 20) have comparatively less marginal discrepancy than those prepared using CAD/CAM. However, the mean of the absolute marginal discrepancy at the incisal edge was higher in the PVC and PVT groups than in the BCM group.

All the three marginal gaps present in groups were clinically acceptable. The highest mean value was reported at the absolute marginal discrepancy at the cervical region of the CAD/CAM-manufactured veneers. For the internal gap, the CBCT analysis reveals almost similar gap ranges (0.1–0.6 mm) between the laminate veneers prepared using three different materials and two fabrication methods.

## 4. Discussion

The marginal discrepancy and internal adaptation of CAD/CAM-milled and 3D-printed laminate veneers depend on the calibration of the equipment and the material utilized for restoration fabrication. Another important aspect is the method used to evaluate marginal and internal adaptation. Some clinicians utilize explorers or 2D radiographs, while researchers use different methods for assessing marginal discrepancy and internal gaps [10,23,24]. The use of stereomicroscopes, scanning electron microscopes, optical microscopes, and profile projectors has been reported. Studies have also noted the use of digitalized images obtained by CBCT and digital cameras, which analyze marginal discrepancy and internal gaps with the help of computer software [19,25,26]. In the present study, CBCT imaging was used to evaluate the variables. This technique allows accurate evaluation in different planes without damaging the prepared specimen. In addition, 3D analysis using CBCT could provide more accurate results by enabling measurements at different points. The major disadvantages of this method include radiation artifacts, technical knowledge requirements, and cost [27]. Nevertheless, studies suggest that this method is accurate and allows a closer view of the different marginal and internal areas of prepared crowns [27,28,29,30].

The value of marginal discrepancy reported in this study was 0.4075 for the CAD/CAM-milled group, 0.31 (PVC), and 0.27 (PVT) for the 3D-printed group. No statistical difference was recorded in marginal discrepancy among the groups. The internal gap reported among the groups was 0.39 for the CAD/CAM group, 0.24 (PVC), and 0.28 (PVT) for the 3D-printed group. Statistical significance (*p* = 0.00) was observed regarding the internal gap among the groups. Accordingly, the null hypothesis that the marginal discrepancy and internal gap will not be influenced by the fabrication method was rejected because of the different measurements between the 3D-printed and milled groups. Similarly, the second null hypothesis that marginal discrepancy and internal gap will not be influenced by the fabrication material utilized was also rejected because of the different measurements between groups, with a minimum in the PVT group and a maximum in the BCM group.

Overall discrepancy showed statistical significance among all the groups (*p* = 0.001). However, no statistical significance was recorded in the absolute marginal discrepancy at the incisal and cervical ends. Similar results were reported in in vitro studies where the fabrication method and materials used significantly influenced the marginal discrepancy and internal adaptation of laminate veneers [18,20,30,31,32]. Contrary to the result of the present study, an in vitro study by Yuce et al. reported no significance of the fabrication method on marginal and internal adaptation [32].

The marginal adaptation values in this study are measurable compared to other studies with similar fabrication methods. Alharabi et al. and Sidhom et al. reported lower marginal discrepancies for 3D-printed laminate veneers compared to CAD/CAM-milled ones [10,33]. A systematic review and meta-analysis indicated a statistically significant mean difference (*p* < 0.05) in favor of 3D-printed veneers over CAD/CAM-milled veneers [1]. The findings of the current study also support the preference for using 3D-printed laminate veneers over CAD/CAM-milled ones. However, almost similar and clinically acceptable marginal discrepancies were reported in the present study, which could be due to variations in the 3D printing models used. For instance, the current study utilized a digital light-processing model, whereas the other two studies utilized stereolithography and inkjet technology to fabricate laminate veneers [11,34].

As far as the internal gap is concerned, statistical significance was reported among the groups measuring lesser internal gap for the 3D-printed veneers. A similar result was reported in recently published in vitro studies [11,28,35]. On the contrary, in vitro studies by Sampaio et al., and Loannidis et al. reported minimal internal discrepancy in veneers fabricated by CAD/CAM milling technique [15,23]. These variations among the studies could be attributed to factors such as tool size limitations, cutting angles, and uneven incisal surface, which can lead to problems during the milling of the intaglio surface.

The increased internal gap (IG) and marginal discrepancy (MD) parameters observed in milled veneers compared to 3D-printed veneers in the current study can be attributed to the inherent differences in fabrication processes and material handling. CAD/CAM milling, used in traditional veneer fabrication, involves the subtractive manufacturing process, which can introduce slight inaccuracies, particularly at fine margins and complex contours [23,35]. Milling tools are often limited by their diameter, which can restrict precision in achieving thin edges and intricate designs, potentially resulting in larger marginal discrepancies [23,35]. In contrast, 3D printing operates as an additive manufacturing method, which allows for higher precision when layering material. This process enables greater control over the final contour and thickness, resulting in a more precise fit with smaller internal gaps and marginal discrepancies [8,11]. Additionally, 3D printing builds veneers layer-by-layer with high accuracy along the entire contour, reducing the risk of dimensional inaccuracies that can occur during the milling process [8,11]. The differences observed in IG and MD parameters underscore the impact of manufacturing methods on fit accuracy. These findings suggest that while both methods are clinically viable, 3D printing may offer greater precision in veneer adaptation and marginal accuracy, which could enhance clinical outcomes.

In this study, laminate veneers were prepared following the butt joint tooth preparation type, as minimal tooth structure is removed to place veneers. In a similar study by Kaur et al. [29], a standard typodont tooth was prepared using the butt joint type. Correct tooth preparation is important to minimize internal and marginal gaps, leading to restoration success. Tooth preparation should include the retention and resistance forms so that the design created facilitates the laboratory fabrication of the restoration. A few studies have reported that decreased restorative material thickness can worsen mechanical properties and increase the chances of material fracture [10,28,33]. However, recent studies reported that materials such as composite-based CAD/CAM (Brilliant Crios) and 3D-printed composite (els-3D Harz) could perform better when fabricated in a thin cross-section as they have higher resilience and greater stress-bearing capacity [35,36]. The post hoc analysis of the current study reported a significantly smaller internal gap of Group PVC when compared to Group BCM.

The current study highlights the importance of a comprehensive approach to endodontic and prosthodontic treatment, encompassing the careful selection of fabrication techniques, materials, and informed digital technologies for evaluation. Clinicians and dental practitioners can enhance their clinical decisions and improve treatment outcomes based on the inferences obtained from this study. These steps will eventually elevate patient-related outcomes and provide a win–win situation. Only a limited number of studies have examined the marginal discrepancy and internal gap of laminate veneers fabricated by CAD/CAM and 3D printers, and a few studies have reported a similar result to the current study [13,30,36,37,38]. However, other studies have not found any significant association among the groups for marginal discrepancy [19,28,32].

While milling typically does not alter the chemical composition of pre-sintered or cured blocks, 3D printing often involves light- or heat-curing steps that can affect material properties. For instance, the degree of polymerization in resin-based materials can vary depending on curing time, intensity, and wavelength in 3D printing, which could potentially affect the final chemical structure and stability. Incomplete curing might lead to residual monomers, which could degrade over time, impacting biocompatibility and longevity [28]. Process parameters such as milling-tool diameter and speed or layer thickness and curing parameters in 3D printing significantly impact mechanical properties. In milling, tool wear or improper feed rates can create micro-fractures in ceramic materials, potentially reducing their fracture toughness and compressive strength. Meanwhile, in 3D printing, layer thickness, printing orientation, and curing method affect the bonding strength between layers. Higher resolution and controlled curing in 3D printing may enhance cohesive strength and overall durability, while improper settings could reduce mechanical integrity [39]. These process-driven factors can ultimately influence the veneers’ strength, wear resistance, and fit accuracy, underscoring the need for optimized settings in both milling and 3D printing to ensure desirable clinical outcomes.

Controlled clinical studies are regarded as the gold standard for assessing the performance of biomaterials and designing aspects of dental prostheses [40]. However, there is currently a lack of in vivo studies and methodologies that can determine the ideal measure of marginal discrepancy necessary to prevent cement dissolution and the penetration or accumulation of oral biofilm [40]. While in vitro studies cannot replace clinical trials, they do offer valuable insights by providing standardized conditions related to preparation design, impression techniques, and operator actions, allowing for the evaluation of individual factors influencing marginal adaptation.

This study has several limitations. First, the study includes measurements taken without considering cementation. Further research on cemented restorations will aid in determining the effect of cement on internal and marginal discrepancies. Second, integrating CBCT data with digital devices is challenging because of incompatible data formats between CBCT and CAD/CAM systems, underscoring the necessity for more research in this area. Thirdly, the unavailability of 3D printing technology for ceramic restorations limits our investigation, as ceramics remain the material of choice for veneers due to their superior esthetic and mechanical properties. Currently, 3D printing technologies primarily support resin-based materials, which may not fully replicate the performance characteristics of ceramics. The absence of 3D-printed ceramic options may have influenced the adaptability and accuracy in simulating clinical scenarios. Additionally, the use of standardized CAD/CAM resin dyes for testing could provide more precise and reproducible results. Utilizing CAD/CAM resin dyes would likely yield results more representative of clinical applications, particularly in color matching and material integrity under stress conditions. However, this was not feasible within the current study parameters, which may have introduced slight variabilities. Further research should focus on clinical trials with different 3D-printed and milled CAD/CAM systems with different ranges and points of adaptation to measure the longevity of restoration.

## 5. Conclusions

Under the limitations of the study, results show that veneers made by VarseoSmile Crown Plus had significantly smaller internal gaps than those made with Brilliant Crios. Additionally, CAD/CAM-milled veneers showed the largest mean difference in absolute marginal discrepancy at the cervical region, indicating a lower marginal accuracy than the other groups.

## Figures and Tables

**Table 1:** Materials utilized in the preparation of laminate veneers.

Group	Materials	Processing Type	Shade	Composition	Manufacturer
PVT	VarseoSmile TriniQ ^a^	Additively manufactured composite resin	A2 Dentin	4,4′-isopropylidenediphenol, ethoxylated and 2-methylprop-2-enoic acid, Benzene acetic acid, alpha–oxo- methyl ester, diphenyl (2,4,6-trimethylbenzoyl) phosphine oxide. (Exact composition not provided by the company.)	Bego Bremen, Germany
PVC	VarseoSmile Crown Plus ^a^	Additively manufactured composite resin	A2 Dentin	Total content of inorganic fillers (particle size 0.7 μm) 30–50 wt%, 4,4′-isopropylidiphenol, ethoxylated and 2-methylprop-2enoic acid, silanized dental glass, methyl benzoylformate, diphenyl (2,4,6-trimethylbenzoyl) phosphine oxide.	Bego, Bremen, Germany
BCM	Brilliant Crios ^b^	Subtractive manufactured composite resin	A2HT14	29.3 wt% cross-linked methacrylates, barium glass (<1.0 μm), 70.7 wt% amorphous silica (<20 nm), and organic pigments such as ferrous oxide or titanium dioxide [20].	Coltene/Whaledent AG, Altstatten, Switzerland

^a^ Bego and ^b^ Coltene brochures.

**Table 2:** Comparing various parameters between the groups.

Variables	Groups	*n*	Mean	Standard. Deviation	95% Confidence Interval for Mean		*p*-Value *
					Lower Bound	Upper Bound	
Marginal discrepancy (MD)	PVT	10	0.2725	0.15159	0.1641	0.3809	0.064
	PVC	10	0.3175	0.11963	0.2319	0.4031	
	BCM	10	0.4075	0.09650	0.3385	0.4765	
	Total	30	0.3325	0.13312	0.2828	0.3822	
Internal discrepancy (ID)	PVT	10	0.2488	0.07937	0.1920	0.3055	0.000
	PVC	10	0.2850	0.08494	0.2242	0.3458	
	BCM	10	0.3975	0.05062	0.3613	0.4337	
	Total	30	0.3104	0.09560	0.2747	0.3461	
Absolute marginal discrepancy (AMD)	PVT	10	0.4500	0.11785	0.3657	0.5343	0.570
	PVC	10	0.4900	0.14870	0.3836	0.5964	
	BCM	10	0.5200	0.16865	0.3994	0.6406	
	Total	30	0.4867	0.14440	0.4327	0.5406	
Overall discrepancy (OD)	PVT	10	0.2843	0.08983	0.2200	0.3485	0.001
	PVC	10	0.3236	0.07657	0.2688	0.3783	
	BCM	10	0.4179	0.03536	0.3926	0.4431	
	Total	30	0.3419	0.08923	0.3086	0.3752	
(AMD-Incisal)	PVT	10	0.4700	0.13375	0.3743	0.5657	0.721
	PVC	10	0.5200	0.16193	0.4042	0.6358	
	BCM	10	0.4600	0.22211	0.3011	0.6189	
	Total	30	0.4833	0.17237	0.4190	0.5477	
(AMD-cervical)	PVT	10	0.4300	0.22136	0.2716	0.5884	0.243
	PVC	10	0.4600	0.22211	0.3011	0.6189	
	BCM	10	0.5800	0.16865	0.4594	0.7006	
	Total	30	0.4900	0.20902	0.4120	0.5680	

* One-way ANOVA; *p*-value < 0.05—statistically significant; PVT: VarseoSmile TriniQ; PVC: VarseoSmile Crown Plus; BCM: Brilliant Crios.

**Table 3:** Post hoc Bonferroni test for pairwise comparison.

Dependent Variable			Mean Difference	Std. Error	*p*-Value *	95% Confidence Interval	
						Lower Bound	Upper Bound
Internal discrepancy (ID)	PVT	PVC	−0.03625	0.03274	0.834	−0.1198	0.0473
		BCM	−0.14875 *	0.03274	0.000	−0.2323	−0.0652
	PVC	BCM	−0.11250 *	0.03274	0.006	−0.1961	−0.0289
Overall discrepancy (OD)	PVT	PVC	−0.03929	0.03181	0.683	−0.1205	0.0419
		BCM	−0.13357 *	0.03181	0.001	−0.2148	−0.0524
	PVC	BCM	−0.09429 *	0.03181	0.019	−0.1755	−0.0131

* *p*-value < 0.05—statistically significant.

## Data Availability

The data that support the findings of this study are available from the corresponding author upon reasonable request.

## Supplementary Materials

The following supporting information can be downloaded at: https://www.mdpi.com/article/10.3390/jfb15110338/s1, Figure S1: showing mean discrepancy of all the mesio-distal and inciso-cervical points for 3Dprinted and milled veneers.
