# Supplementary material for: Marginal Discrepancy and Internal Fit of 3D-Printed Versus Milled Laminate Veneers: An In Vitro Study
Source: J Funct Biomater. 2024 Nov 11;15(11):338. doi: 10.3390/jfb15110338 (PMC11595794; doi:10.3390/jfb15110338)
Supplement: Supplementary file 1 [file jfb-15-00338-s001.zip › jfb-3275260-Supplementary material.pdf]

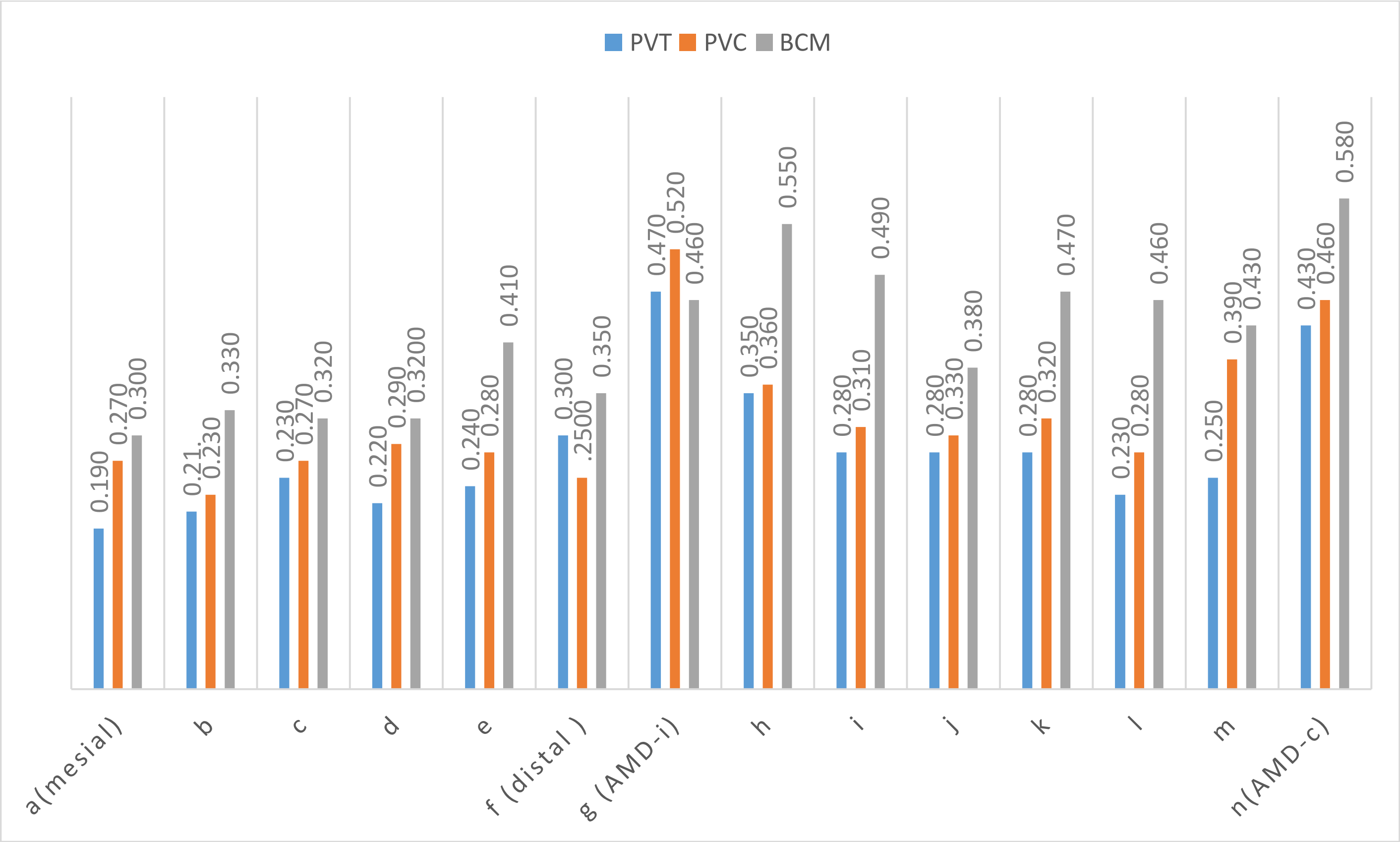

Graph –S1-showing mean discrepancy of all the mesio-distal and inciso-cervical points for 3Dprinted and milled veneers
